# Bismuth Vanadate Capable of Driving One-Step-Excitation Photocatalytic Overall Water Splitting

**DOI:** 10.1021/jacs.4c18733

**Published:** 2025-03-24

**Authors:** Hao Wu, Songying Qu, Yun Hau Ng

**Affiliations:** † Macau Institute of Materials Science and Engineering (MIMSE), Faculty of Innovation Engineering, Macau University of Science and Technology, Taipa 999078, Macau SAR; ‡ School of Energy and Environment, City University of Hong Kong, Kowloon Tong 999077, Hong Kong SAR; § Center for Renewable Energy and Storage Technologies (CREST), Physical Science and Engineering (PSE) Division, King Abdullah University of Science and Technology (KAUST), Thuwal 23955-6900, Saudi Arabia; ∥ Clean Energy Research Platform (CERP), Physical Science and Engineering (PSE) Division, King Abdullah University of Science and Technology (KAUST), Thuwal 23955-6900, Saudi Arabia

## Abstract

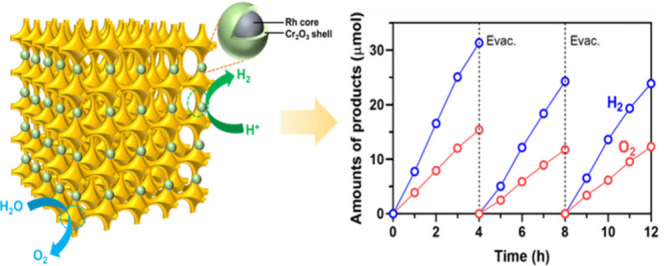

Solar-driven one-step-excitation overall water splitting is one of the most straightforward ways of achieving scalable solar hydrogen production. Despite its advantage in visible-light response and stability, the pristine bismuth vanadate (BiVO_4_) alone is not capable of achieving overall water splitting due to its conduction band energy level being insufficient for the proton reduction reaction, further limited by the severe charge recombination. Here, we demonstrate the three-dimensionally ordered macroporous BiVO_4_ with a carefully enlarged bandgap of 2.58 eV and a conduction band energy of −0.49 eV versus normal hydrogen electrode at pH 7.0. On loading Rh/Cr_2_O_3_ as a hydrogen evolution cocatalyst, the stochiometric evolution of hydrogen and oxygen in a recyclable manner is accomplished under visible light with an apparent quantum yield of 0.47% at 400 nm. Further study by Kelvin probe force microscopy reveals an intensified internal electric field with a downward band bending from the interconnection part to the internal wall of the periodic porous structure upon loading the cocatalysts. Our findings transform BiVO_4_ from a typical water oxidation photocatalyst into a new paradigm for single-component photocatalysts for overall water splitting.

Photocatalytic overall water splitting by a single particulate semiconductor component is a simple and cost-effective means of producing green hydrogen (H_2_) on a large scale.^1^ Developing an efficient and cost-competitive photocatalytic overall water splitting system requires narrow-bandgap photoactive materials capable of driving the reaction under visible light.^2^ Metal sulfide photocatalysts have shown promising visible-light-driven overall water splitting activities but are prohibited by their self-oxidation of lattice sulfide ions, leading to photocorrosion.^3^ Strategies, such as defect engineering and hybridization of the S-3*p* orbital with the O-2*p* orbital (oxysulfide photocatalyst), could prevent sulfide ions from self-oxidation.^4,5^ However, there is still a limited number of reports using visible-light-responsive metal oxide as the only photoactive component for one-step-excitation photocatalytic overall water splitting to produce H_2_ and oxygen (O_2_).

Bismuth vanadate (BiVO_4_) has been perceived as a promising O_2_ evolution photocatalyst owing to its simple structure, narrow bandgap, and relatively good stability.^6,7^ Nevertheless, there is limited research on photocatalytic H_2_ production of BiVO_4_ due to its inappropriate conduction band (CB) located at a more positive potential than the proton reduction potential (i.e., CB > 0 eV vs normal hydrogen electrode (NHE) at pH 0).^8^ Also, the slow transfer of photoinduced electron and hole pairs to redox sites, along with high surface reaction barriers, is a bottleneck problem of BiVO_4_. To date, there is a limited successful example of using BiVO_4_ photocatalyst (Bi_1–*x*_In_*x*_V_1–*x*_Mo_*x*_O_4_) for one-step-excitation overall water splitting through phase-transition-induced band edge engineering by dual doping with In and Mo.^9^ More recently, Askarova et al. also visualized O_2_ and H_2_ evolutions on {110} and {010} lateral facets at a single truncated bipyramidal microcrystal of phosphorus-doped BiVO_4_, respectively.^10^ Following these works, there have been very limited studies on BiVO_4_ for one-step-excitation overall water splitting, likely restrained by the lack of an approach that can unlock the thermodynamic and charge carrier dynamic potentials of BiVO_4_.^11^

A highly ordered three-dimensional macroporous (3DOM) BiVO_4_ photocatalyst with high crystallinity connected by the ultrathin internal wall demonstrated a quantum confinement effect.^12^ The quantum confinement has increased the CB position of the BiVO_4_ photocatalyst, enabling H_2_ evolution activity with a yield of 13 μmol h^–1^ under visible light in the presence of sacrificial agents. Despite the uplifted CB being sufficient to reduce protons to produce H_2_, the 3DOM BiVO_4_ photocatalyst failed to produce stoichiometric H_2_ and O_2_ from pure water due to higher thermodynamic barriers and stronger charge recombination. Therefore, loading a cocatalyst may be a critical measure to improve the photocatalytic overall water splitting activity of 3DOM BiVO_4_.

Among various cocatalysts, Rh/CrO_*x*_ has been widely applied in photocatalytic water splitting studies over a wide range of semiconducting systems such as oxysulfide,^5^ oxynitride,^13^ and ternary oxide.^1,14^ Here, upon loading with H_2_ evolution cocatalyst of Rh/Cr_2_O_3_ and fine-tuning the reaction conditions, we demonstrate a one-step-excitation photocatalytic overall water splitting, which evolves H_2_ and O_2_ in a stoichiometric ratio using 3DOM BiVO_4_ under visible light. We also elucidate the intensified internal electric field between the internal wall and the interconnection part of 3DOM BiVO_4_ upon cocatalyst loading through Kelvin probe force microscopy (KPFM).

The synthesis of 3DOM BiVO_4_ and the subsequent deposition of Rh/Cr_2_O_3_ cocatalyst are detailed in the Experimental Section of Supporting Information.^15^ Upon loading Rh/Cr_2_O_3_, the 3DOM BiVO_4_ achieved the stoichiometric evolution of H_2_ and O_2_ under visible light (>420 nm) during a 12 h reaction (Figure 1a). Note that the optimal pH of the reaction solution and the cocatalyst loading amount were determined to be 7.0 and 2.0 wt % Rh with 1.0 wt % Cr_2_O_3_ (Table 1, entries 4–9), respectively. The decoration of Cr_2_O_3_, which can prevent the back reaction of O_2_ reduction on the Rh active sites, is indispensable to achieving the overall water splitting activity.^16^ During the second run of the test, the performance was slightly decreased, which is often observed for most overall water splitting systems.^11,13^ The performance at the third run of the test was the same as or even slightly better than that of the second cycle, indicating a relatively stable system. Besides, the inductively coupled plasma (ICP) analysis of the reaction solution suggests negligible Cr, Rh, Bi, and V, excluding the parasitic reaction of Rh/Cr_2_O_3_/3DOM BiVO_4_. Notably, the wavelength-dependent apparent quantum yields (AQYs) determined for Rh/Cr_2_O_3_/3DOM BiVO_4_ were consistent with the UV–vis result acquired from 3DOM BiVO_4_ (Figure 1b). The AQY value for Rh/Cr_2_O_3_/3DOM BiVO_4_ at 400 nm was achieved at 0.47 ± 0.02%.

**Figure 1:**
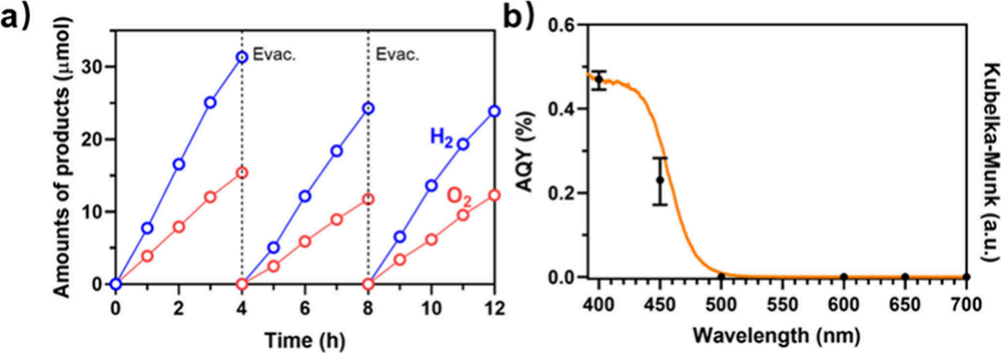
(a) Time course of overall water splitting under visible-light irradiation (300 W, >420 nm) by the Rh/Cr_2_O_3_/3DOM BiVO_4_ system. (b) The wavelength-dependent apparent quantum yield (AQY) and the UV–vis diffuse reflectance spectrum (DRS) of the Rh/Cr_2_O_3_/3DOM BiVO_4_ sample.

**Table 1:** Overall Water Splitting under Visible-Light Irradiation by the 3DOM BiVO_4_ System^a^

					activity (μmol)	
entry	Cocatalyst	Scavenger	photocatalyst	pH	H_2_	O_2_
1	–	–	3DOM BiVO_4_	7.0	0	0
2	Rh/Cr_2_O_3_^b^	–	Irregular BiVO_4_	7.0	0	0
3	Rh/Cr_2_O_3_^b^	–	Platelike BiVO_4_	7.0	0	0
4	Rh/Cr_2_O_3_^b^	–	3DOM BiVO_4_	7.0	31.3	15.4
5	Rh^c^	–	3DOM BiVO_4_	7.0	17.7	1.8
6	Rh^d^	–	3DOM BiVO_4_	7.0	23.3	0
7	Rh^e^	–	3DOM BiVO_4_	7.0	9.6	0
8	Rh^d^	–	3DOM BiVO_4_	4.0	5.7	0
9	Rh^d^	–	3DOM BiVO_4_	9.0	9.6	2.8
10	–	Na_2_SO_3_	3DOM BiVO_4_	9.0	38.8	0
11	–	Fe(NO_3_)_3_	3DOM BiVO_4_	7.0	0	159.0

a Conditions: light source, 300 W Xe lamp (λ > 420 nm); photocatalyst (0.05 g) in water, H_2_SO_4_ or NaOH (aq) (50 mL); continuous reaction for 4 h.

b 2.0 wt % of Rh and 1.0 wt % of Cr_2_O_3_.

c 1.0 wt % of Rh.

d 2.0 wt % of Rh.

e 3.0 wt % of Rh.

To investigate the underlying mechanism, we first established, by means of the control experiments using the BiVO_4_ counterparts with commonly seen irregular and platelike morphologies (Table 1, entry 2 and entry 3), that the one-step-excitation overall water splitting is indeed owing to the periodically ordered porous structure of 3DOM BiVO_4_. From the transmission electron microscopy (TEM) image (Figure S1a,b), the 3DOM BiVO_4_ showed the ultrathin (<10 nm) and crystalline internal wall, consistent with the literature.^12^ The scanning electron microscopy (SEM) images of irregular and platelike BiVO_4_ show their bulk properties (Figure S2a,b). UV–vis and ultraviolet photoelectron spectroscopy (UPS) measurements were performed to determine the band energy positions (Figure 1b and Figure 2).^12,17^ The bandgap of 3DOM BiVO_4_ was determined to be 2.58 eV with the conduction band and valence band at −0.49 and 2.09 eV vs NHE at pH 7.0 (inset of Figure 2), see detailed calculation in the Experimental Section of Supporting Information. In contrast to the bandgap energy of ∼2.40 eV for irregular and platelike BiVO_4_ counterparts, as widely reported,^12^ the quantum confinement effect arising from the 3DOM structure enlarged the bandgap of BiVO_4_ with an uplifted CB, which is thermodynamically favorable for a proton reduction reaction to produce H_2_. In the presence of Na_2_SO_3_ as hole scavengers, the 3DOM BiVO_4_ produced 38.8 μmol of H_2_ for 4 h (Table 1, entry 10), while in the presence of Fe(NO_3_)_3_ as electron scavengers, 159.0 μmol of O_2_ was produced (Table 1, entry 11). The parent 3DOM BiVO_4_ without cocatalyst loading and sacrificial agents showed negligible water splitting activity due to the high thermodynamic reaction barrier and severe charge recombination (Table 1, entry 1), requiring the cocatalyst to facilitate the overall water splitting reaction.

**Figure 2:**
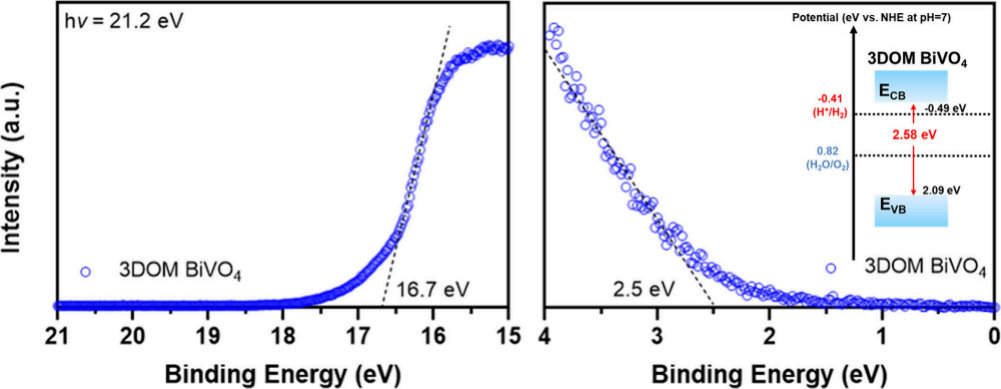
UPS spectra with cutoff region (left) and valence band edge region (right) of platelike and 3DOM BiVO_4_.

A loading of 2.0 wt % Rh on the BiVO_4_ photocatalyst offered the highest H_2_ production activity from water splitting with higher loading (3.0 wt %) decreasing the performance (Table 1, entries 5–7), likely caused by parasitic light absorption of the Rh cocatalyst that competes with the BiVO_4_ photocatalyst.^18^ In the absence of Cr_2_O_3_, no photocatalytic O_2_ evolution was observed, and only a small amount of O_2_ was detected for the BiVO_4_ with 1.0 wt % Rh, which has been reported previously and could potentially be ascribed to the strong O_2_ reduction reaction (ORR) capability of Rh,^14^ while the Cr_2_O_3_ shell could suppress ORR by preventing the access of produced O_2_ to the Rh surface (Table 1, entry 4).^16^ We also studied the photocatalytic activity of BiVO_4_ as a function of pH with 2.0 wt % Rh (Table 1, entries 8 and 9). The activity was found to vary at the different pH values, and activities were lower at pH 4.0 and pH 9.0 than at pH 7.0. We note that the band edges of semiconducting oxides have the same pH dependence as the redox potentials of the water splitting.^19,20^ The pH-dependent photocatalytic water splitting performance is likely due to the ionization potential and the electron affinity of Rh/Cr_2_O_3_/3DOM BiVO_4_ photocatalyst relative to the redox potentials of water splitting.^18,21^

The BiVO_4_ photocatalysts with or without cocatalyst loading showed comparable X-ray diffractometer (XRD) patterns (Figure S3), suggesting the monoclinic crystal structure of BiVO_4_.^22^ After the photodeposition of Rh/Cr_2_O_3_, no obvious diffraction peaks ascribed to Rh and Cr_2_O_3_ were observed, resulting from their small size and insignificant amount. The high-resolution transmission electron microscopy (HRTEM) images show that the cocatalyst particles were evenly spread on the surface of BiVO_4_ (Figure S4a,b) and identified to be Rh and Cr by the corresponding energy-dispersive X-ray spectroscopy (EDX) mapping images (Figure S5). In line with the literature, the Rh particles with a size between 3.0 and 8.0 nm were observed, while the Cr_2_O_3_ shell was approximately 1.5 nm in thickness.^5^ X-ray photoelectron spectroscopy (XPS) results disclosed that the Rh and Cr species on the surface of BiVO_4_ had valence states attributable to metallic Rh and Cr_2_O_3_ (Figure S6), respectively.

KPFM measurements further unravel the electrical potential distribution in the Rh/Cr_2_O_3_-deposited 3DOM BiVO_4_ photocatalyst by spatially resolved surface photovoltage.^23−25^Figure S7a,b depicts the topography of the 3DOM BiVO_4_ samples with and without Rh/Cr_2_O_3_ cocatalyst loading, showing an insignificant difference. The KPFM image and the corresponding surface potential curve represented by the BiVO_4_ macropore region, as shown in Figure 3a,c, suggest the internal electric field and an upward band bending from the internal wall to the interconnection part of the 3DOM BiVO_4_. The surface potential at the interconnection part is ∼25 mV lower than the internal wall, likely arising from their well-distributed lattice planes as reported in the literature.^12^ Upon loading the Rh/Cr_2_O_3_ cocatalyst, the surface potential difference between the interconnection and the internal wall (Δφ) is increased to ∼50 mV (Figure 3b,d). The KPFM potential line scans at other spots of pristine 3DOM BiVO_4_ and Rh/Cr_2_O_3_/3DOM BiVO_4_ were also performed (Table S1), showing a similar trend of the surface potential difference over different locations. It indicates that the band bending within the space charge region near the interconnection surface of 3DOM BiVO_4_ steepened, while that near the internal wall surface flattened, thus intensifying the internal built-in electric field between the interconnection and the internal wall (Figure 4a,b). Hence, in addition to the accelerated reaction kinetics and the prevented back reaction, the spatial charge separation of 3DOM BiVO_4_ could also be improved after the loading of the Rh/Cr_2_O_3_ cocatalyst on its surface, accumulating more electrons in the internal wall and extracting the holes near the interconnection part more effectively, thus enabling the Rh/Cr_2_O_3_/3DOM BiVO_4_ photocatalyst for one-step-excitation photocatalytic water splitting that cannot be achieved by the pristine 3DOM BiVO_4_ counterpart (Figure 4c,d).

**Figure 3:**
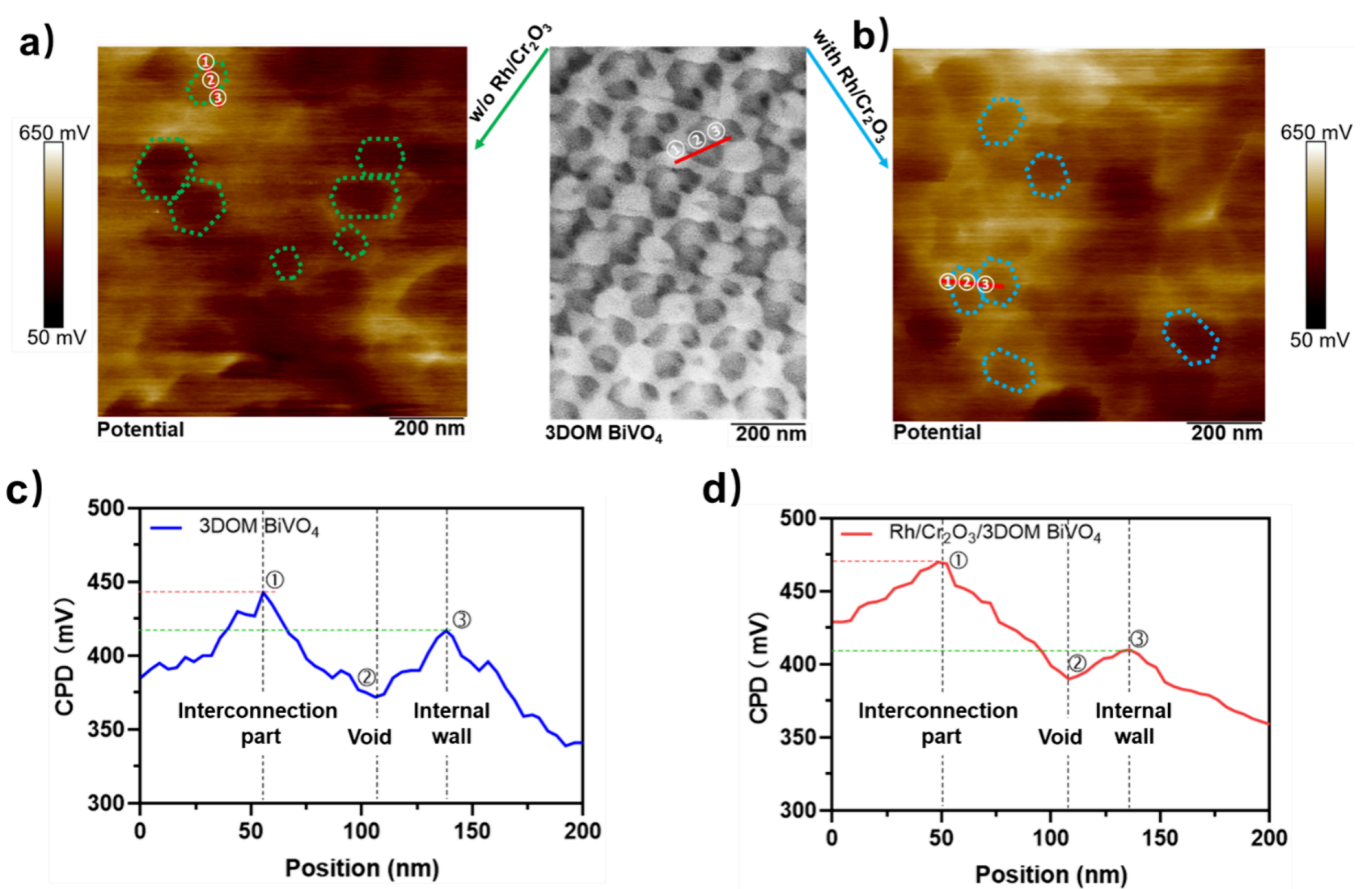
KPFM surface potential maps of (a) 3DOM BiVO_4_ and (b) Rh/Cr_2_O_3_/3DOM BiVO_4_. The inset between a and b is the electron microscopy image of 3DOM BiVO_4_. The surface potential changes from the interconnection (①), the void (②), and the internal wall (③) cross the region marked by the red line in the corresponding KPFM maps of (c) 3DOM BiVO_4_ and (d) Rh/Cr_2_O_3_/3DOM BiVO_4_.

**Figure 4:**
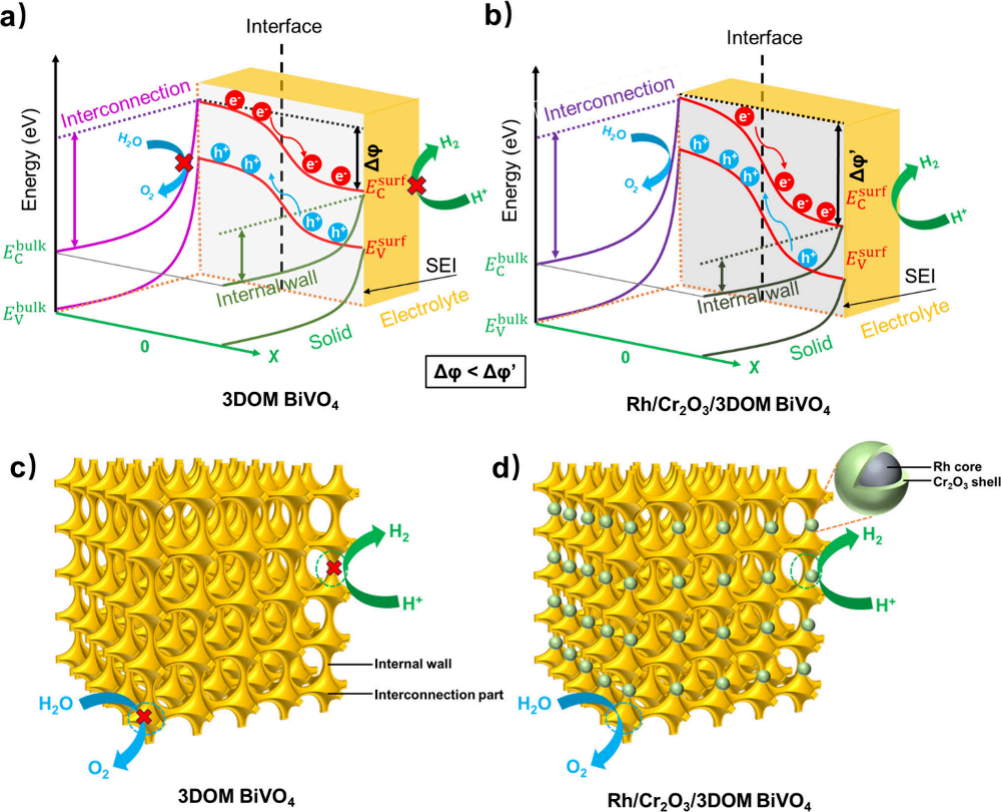
Energy diagram demonstrating the internal electric field and the charge transfer directions at the junction of the interconnection part and the internal wall of (a) 3DOM BiVO_4_ and (b) Rh/Cr_2_O_3_/3DOM BiVO_4_. SEI represents the semiconductor and electrolyte interface. Schematic illustration of one-step excitation photocatalytic water splitting by (c) 3DOM BiVO_4_ and (d) Rh/Cr_2_O_3_/3DOM BiVO_4_.

In summary, overall photocatalytic water splitting via one-step excitation under visible light was realized by 3DOM BiVO_4_. The 3DOM BiVO_4_ showed an upshifted CB position compared to its bulk counterparts, enabling it toward proton reduction from water splitting. KPFM results unraveled that incorporating the Rh/Cr_2_O_3_ cocatalyst further adjusted the internal electric field and steepened the upward bind bending from the internal wall to the interconnection part of 3DOM BiVO_4_. Loading the Rh/Cr_2_O_3_ cocatalyst, stochiometric evolution of H_2_ and O_2_ in a ratio of 2:1 with an AQY of 0.47% at 400 nm was achieved. Further attempts at doping, cocatalyst modification, and dimension control over the presented photocatalyst system are expected to increase the efficiency. Although this present efficiency is still low, this study extended the BiVO_4_ photocatalyst from water oxidation to one-step excitation overall water splitting by nanostructuring and cocatalyst, opening new opportunities for research into efficient and scalable H_2_ production from solar energy.

## Supporting Information Available

Experimental details, SEM images, TEM images, XRD patterns, HAADF-EDX mapping, XPS spectra, and KPFM surface potential table (PDF)
