# Supplementary material for: Bismuth Vanadate Capable of Driving One-Step-Excitation Photocatalytic Overall Water Splitting
Source: J Am Chem Soc. 2025 Mar 24;147(13):10829–33. doi: 10.1021/jacs.4c18733 (PMC11969541; doi:10.1021/jacs.4c18733)
Supplement: Supplementary file 1 — ja4c18733_si_001.pdf [file ja4c18733_si_001.pdf]

Supporting Information

Bismuth Vanadate Capable of Driving One-step-excitation Photocatalytic Overall Water Splitting

Hao Wu,[a]* Songying Qu,[a,b] and Yun Hau Ng[b,c,d]*

[a]Macau Institute of Materials Science and Engineering (MIMSE), Faculty of Innovation Engineering, Macau University of Science and Technology, Taipa 999078, Macau, SAR;

Email: wuhao@must.edu.mo;

[b] School of Energy and Environment, City University of Hong Kong, Kowloon Tong, Hong Kong 999077, SAR;

Email: yunhau.ng@cityu.edu.hk;

[c] Center for Renewable Energy and Storage Technologies (CREST), Physical Science and Engineering (PSE) Division, King Abdullah University of Science and Technology (KAUST), Thuwal 23955-6900, Saudi Arabia

[d] Clean Energy Research Platform (CERP), Physical Science and Engineering (PSE) Division, King Abdullah University of Science and Technology (KAUST), Thuwal 23955-6900, Saudi Arabia

EXPERIMENTAL SECTION

Preparation of Photocatalysts. The 3DOM $BiVO_4$ photocatalysts were synthesized through a PMMA-temple method, according to a previous report.[1] In brief, 4.87 g $Bi(NO_3)_3{\bullet}5H_2O$ was added to a mixture solution of 6 mL methanol and 3 mL ethylene glycol. The solution was vigorously stirred for 1 h until a transparent solution was obtained. Another mixture solution was simultaneously prepared, containing 1.17 g NH_4NO_3, 6 mL H_2O, 1.75 g L-ascorbic acid, and 1.6 mL nitric acid (69 wt%). The above two solutions were mixed at 70 ^oC with stirring for 1 h. The as-prepared PMMA (2.0 g) was further soaked into the mixture solution for 3 h after naturally cooling down to room temperature. The prepared powders were obtained by suction filtration and dried in the air overnight. Finally, the as-prepared samples were annealed in a tube furnace at 300 ^oC for 2 h and further heated to 450 ^oC for 4 h. The ramping rate of heating is 1 ^oC min^{-1}. The synthesis of PMMA microspheres was adapted from the previous reports.[2,3]

Characterizations. X-ray diffraction (XRD) patterns were acquired by a Phillips X'pert Multipurpose X-ray Diffraction System (MPD) at room temperature using Cu Kα radiation (λ = 1.54184 Å) with a potential of 45 kV and a current of 40 mA. The morphologies were acquired with scanning electron microscopy (FEI SEM450) and high-resolution transmission electron microscopy (HRTEM, CM200 Philips). X-ray photoelectron spectroscopy (XPS) was performed by a Thermo Scientific (ESCALAB220i-XL) instrument with a monochromatic Al Kα X-ray source at 1486.68 eV. Carbon 1*s* peak at 284.6 eV was used to calibrate the XPS data. A ThermoFisher Nexsa spectrometer with monochromatized He-I radiation at 21.2 eV in an ultra-high vacuum was used to conduct ultraviolet photoelectron spectroscopy (UPS) characterizations. Diffuse reflectance spectroscopy (DRS) data were obtained by a UV-Vis-NIR spectrometer equipped with an integrating sphere (UV-3600, Shimadzu). Kelvin probe force microscope (KPFM) measurements were carried out by Bruker Dimension ICON under ambient conditions using PeakForce tapping mode (PeakForce-KPFM-AM). The surface topology was acquired by Bruker's proprietary PeakForce tapping modes in the first pass and the surface potential was obtained in the second pass using the lift mode for surface potential imaging. The scanning tip for all the sample measurements was Pt/Ir-coated SCM-PIT probe.[1–3] The lift height was fixed at 50 nm for all the tests to achieve the highest signal-to-noise ratio for the surface potential imaging. Also, the KPFM measurements were repeated four times at different spots for a fair comparison of contact potential differences (CPDs) between the pristine 3DOM $BiVO_4$ and the Rh/Cr_2O_3/3DOM $BiVO_4$ samples.

Photocatalytic Reactions for Overall Water Splitting. The as-prepared 3DOM $BiVO_4$ powders (50 mg) were dispersed in an enclosed quartz reactor with a Pyrex window containing 50 ml H_2O. Subsequently, the $RhCl_3$ with the desired amount (1.0, 2.0, or 3.0 wt%) and 50 mM Na_2SO_3 were added to the reactor. The reactor was sealed and purged with pure N_2 for 30 min to remove the gas impurities. The photodeposition of Rh cocatalyst was carried out under visible-light irradiation (> 420 nm) for 2 h. In the following step, the 1.0 wt% K_2CrO_4 was added to the reaction solution and irradiated under visible light for another 2 h. After the deposition, the reaction solution was extracted, and the cocatalyst-loaded 3DOM $BiVO_4$ photocatalysts were thoroughly washed with H_2O three times. The light source was provided by a 300 W Xenon lamp (Newport) equipped with a cut-off filter (> 420 nm). The pH of the reaction solution was adjusted by adding diluted H_2SO_4 or NaOH. A Newport optical power meter calibrated the incident light intensity to be 200 mW cm^{-2}. The temperature of the reaction solution was maintained at 25 ^oC by a water circulation system. Prior to the light excitation, the reaction the reaction suspension was purged with N_2 gas for 30 min removing all the air present in the reactor. The produced H_2 and O_2 were quantified by gas chromatography (TCD, Shimadzu GC-8A). We note that a syringe was used for gas sampling. To minimize air contamination, the gas-tight syringe (*i.e.*, a glass syringe with a

PTFE seal) was flushed with N_2 gas before each sampling; 120% of the target volume of the gas product was drawn by the syringe and the excess 20% of the gas volume was expelled from the syringe before sampling; the plunger of the syringe was pulled/pushed slowly to draw/expel gas product; the gas product was immediately analyzed upon sampling; ambient air was periodically sampled with the same syringe for quantification.

Apparent Quantum Yield Measurements. The apparent quantum yield (AQY) was determined using a 300 W Xe lamp (Newport) with bandpass filters (Edmund Optics) having center wavelengths of 400, 450, 500, 550, 600, 650, and 700 nm (full width at half-maximum = 15 nm). The incident light intensity at each wavelength was determined by a Newport optical power meter. The AQY of the photocatalytic overall water splitting reaction was calculated using the following equation:

$$AQY = \frac{2 \times the\ number\ of\ produced\ H_2\ molecules}{the\ number\ of\ incident\ photons} \times 100\%$$

Determination of Band Energy Levels. The conduction band (E_{CB}) and valence band (E_{VB}) energy positions were determined by UV-vis DRS and UPS results. The work function (φ) was determined by the difference between the binding energy of the secondary electron cut-off and the incident photon energy (21.2 eV). For the 3DOM $BiVO_4$, the cut-off binding energy is 16.7 eV as determined by the intersection of the linear extension of the spectrum and the baseline. Hence, the φ of 3DOM $BiVO_4$ was determined to be 4.5 eV (φ = 21.2 – 16.7), which means that the Fermi level (E_F) is -4.5 eV vs. vacuum level (E_{VAC}). The intersection of the baseline with the linear extension of the UPS spectra near the Fermi edge was used to determine the difference between E_{VB} and E_F. The 3DOM $BiVO_4$ has an E_F-E_{VB} = 2.5 eV. Therefore, its E_{VB} = -4.5 eV – 2.5 eV = -7.0 eV vs. E_{VAC}. In other words, the E_{VB} of 3DOM $BiVO_4$ is 2.09 eV vs. NHE at pH = 7.0. Combining with the UV-vis DRS result, the E_{CB} of 3DOM $BiVO_4$ was calculated to be -0.49 eV vs. NHE at pH 7.0 (E_{CB} = E_{VB} – E_g)

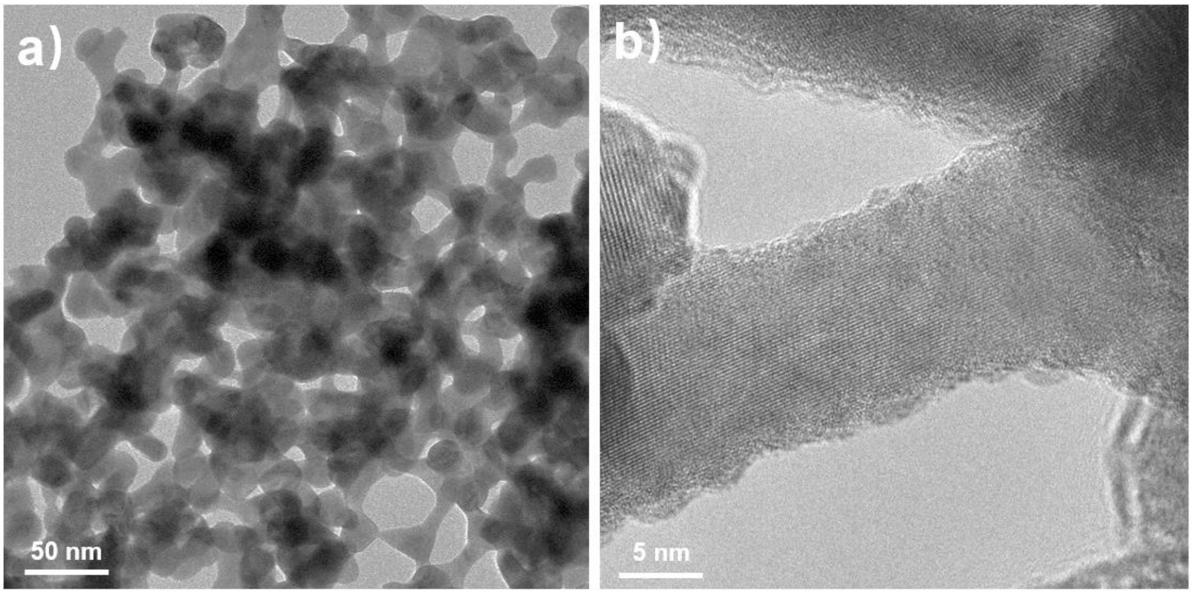

Figure S1. a) TEM image and b) HRTEM image of pristine 3DOM $BiVO_4$ sample.

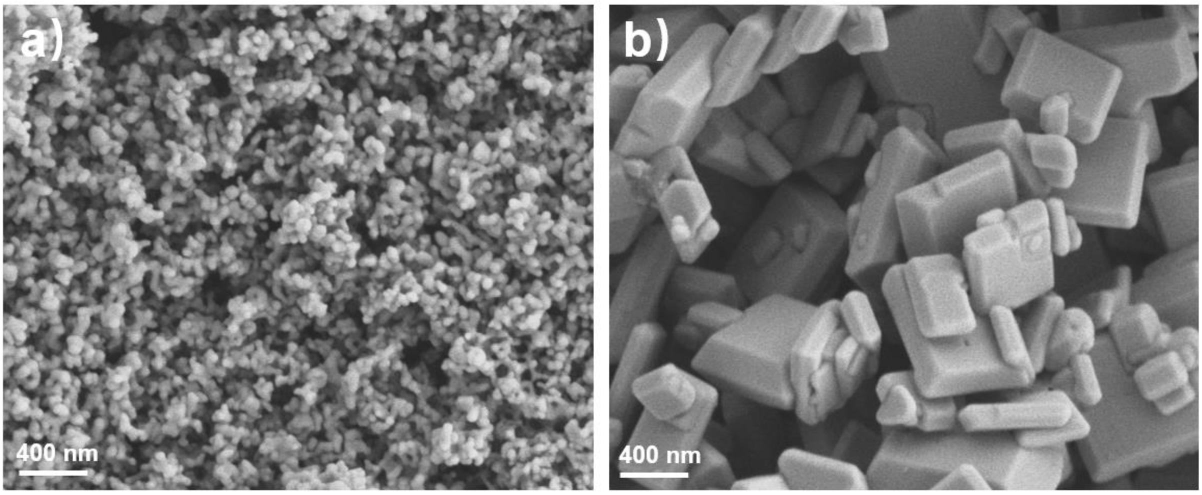

Figure S2. SEM images of a) irregular and b) platelike $BiVO_4$ samples.

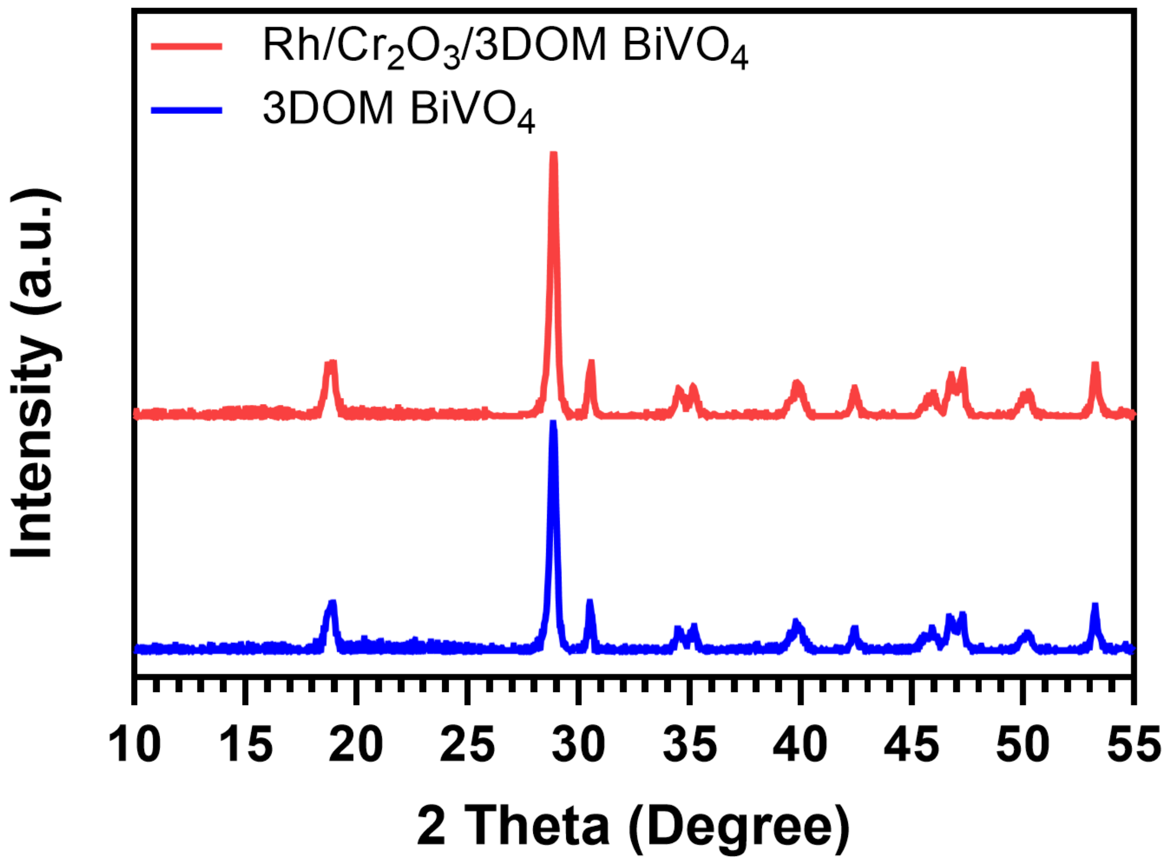

Figure S3. The XRD patterns of 3DOM $BiVO_4$ samples with and without Rh/Cr_2O_3 cocatalyst loading.

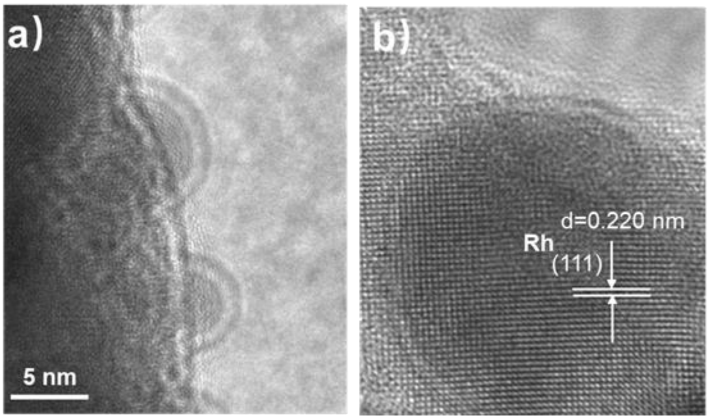

Figure S4. a,b) HRTEM images of $Rh/Cr_2O_3/3DOM\ BiVO_4$ sample at different magnifications.

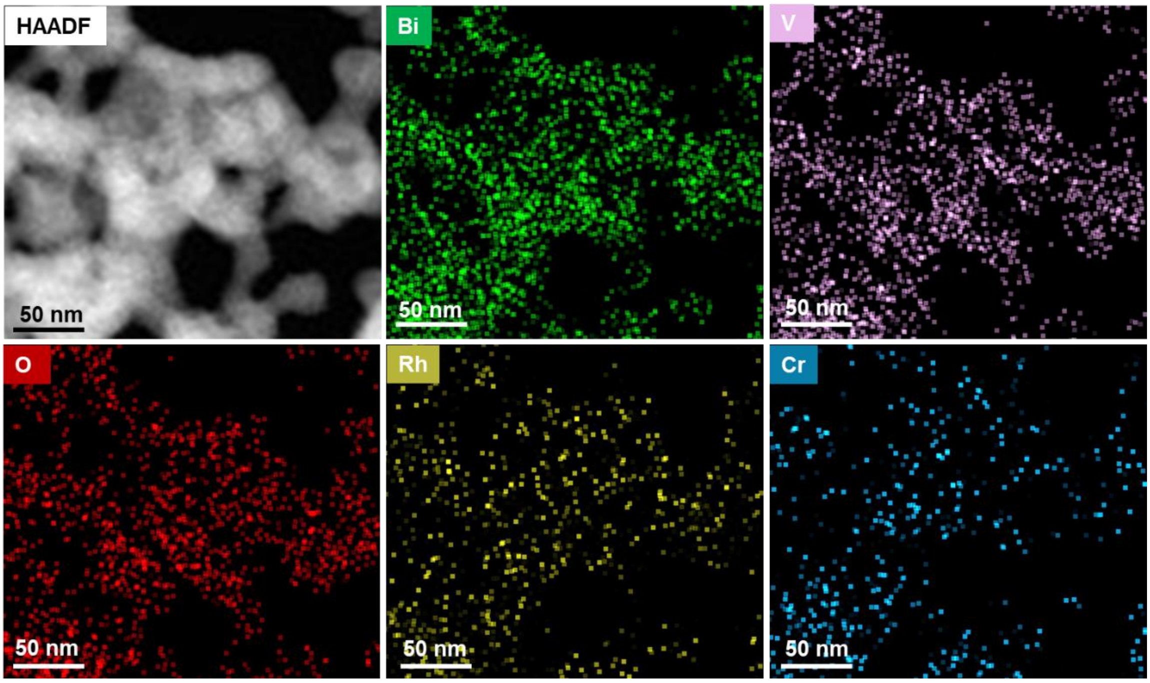

Figure S5. HAADF and the corresponding mapping images of Bi, V, O, Rh, and Rh for the Rh/Cr_2O_3/3DOM $BiVO_4$ sample.

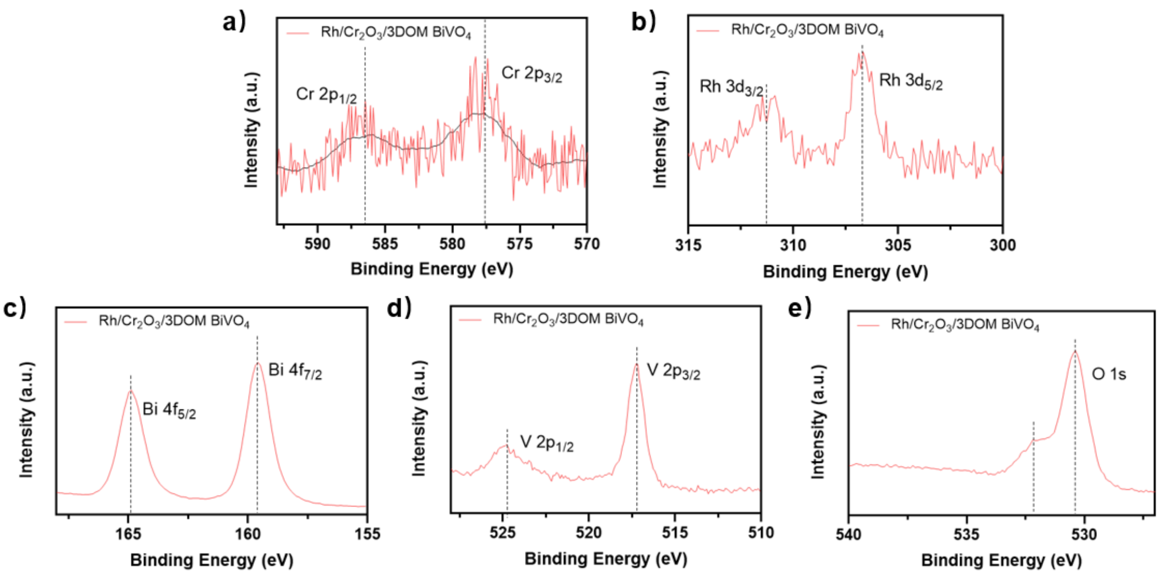

Figure S6. XPS spectra of a) Cr 2p, b) Rh 3d, c) Bi 4f, d) V 2p, and e) O 1s for the $Rh/Cr_2O_3/3DOM\ BiVO_4$ sample.

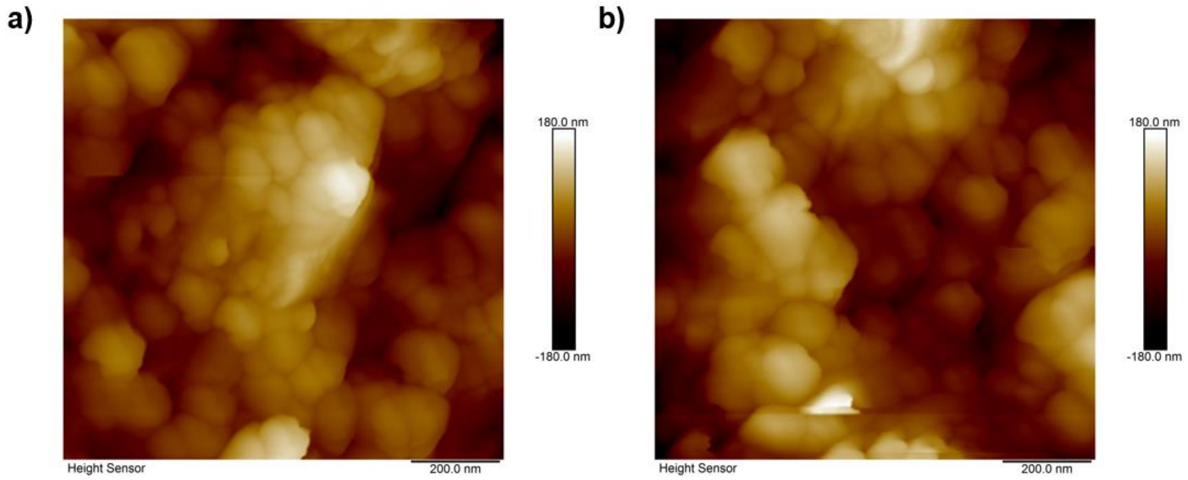

Figure S7. The topography maps of a) 3DOM $BiVO_4$ and b) Rh/Cr_2O_3/3DOM $BiVO_4$.

Table S1. The Surface Potential Difference over Different Locations of 3DOM $BiVO_4$ and $Rh/Cr_2O_3/BiVO_4$ Samples

Location	without Rh/Cr_2O_3 (mV)	with Rh/Cr_2O_3 (mV)
Spot 1	28	51
Spot 2	32	55
Spot 3	21	49

References

(1) He, T.; Zhao, Y.; Benetti, D.; Moss, B.; Tian, L.; Selim, S.; Li, R.; Fan, F.; Li, Q.; Wang, X.; Li, C.; Durrant, J. R. Facet-Engineered $BiVO_4$ Photocatalysts for Water Oxidation: Lifetime Gain Versus Energetic Loss. *J. Am. Chem. Soc.* 2024, *146* (39), 27080–27089.

(2) Zhu, J.; Pang, S.; Dittrich, T.; Gao, Y.; Nie, W.; Cui, J.; Chen, R.; An, H.; Fan, F.; Li, C. Visualizing the Nano Cocatalyst Aligned Electric Fields on Single Photocatalyst Particles. *Nano. Lett.* 2017, *17* (11), 6735–6741.

(3) Kaja, K.; Assoum, A.; De Wolf, P.; Piquemal, F.; Nehmee, A.; Naja, A.; Beyrouthy, T.; Jouiad, M. 3D Imaging and Quantitative Subsurface Dielectric Constant Measurement Using Peak Force Kelvin Probe Force Microscopy. *Adv. Mater. Interfaces* 2024, *11* (2), 2300503.
